# Correlates of Psychological Distress Among Pakistani Adults During the COVID-19 Outbreak: Parallel and Serial Mediation Analyses

**DOI:** 10.3389/fpsyg.2021.647821

**Published:** 2021-03-31

**Authors:** Farzana Ashraf, Gull Zareen, Aasia Nusrat, Amna Arif, Mark D. Griffiths

**Affiliations:** ^1^Department of Humanities, COMSATS University Islamabad, Lahore, Pakistan; ^2^Special Education Department, Government of the Punjab, Lahore, Pakistan; ^3^Laboratory of Psychology and Neurocognition, University of Grenoble Alpes, Grenoble, France; ^4^Department of Education, University of Management and Technology, Lahore, Pakistan; ^5^Department of Psychology, Nottingham Trent University, Nottingham, United Kingdom

**Keywords:** psychological distress, obsessions about COVID-19, meaning in life, satisfaction with life, Pakistani adults

## Abstract

**Objective:** The global outbreak of COVID-19 has greatly affected individual's lives around the world and resulted in various negative psychological consequences. During the pandemic, reflection on and attention to COVID-19 may help in dealing with its symptomology but frequent and persistent thoughts about the situation can be unhealthy. The present study examined the direct and indirect associations between obsession concerning COVID-19, psychological distress, life satisfaction, and meaning in life.

**Design:** This mediation study presents a primary analysis of normative data collected after the initial outbreak of COVID-19 in Pakistan. Parametric bootstrapping was used to test the mediation models of subjective well-being, the extent of the effect, and meaning in life as parallel and serial mediators concerning the associations between COVID-19 obsession and psychological distress measures.

**Setting:** A sample of 1,002 adults (45% men and 55% women) were recruited utilizing an online survey between April to May 2020. They were aged between 19 and 45 years (*M* = 24.30, *SD* = 7.29) and normalized on population characteristics.

**Results:** Two out of three mediators in parallel mediation fully mediated the relationship between obsession and psychological distress (total effect = 0.443, *SE* = 0.050, *p* < 0.0001) illustrating that high-level obsessions were associated with low levels of satisfaction with life and presence of meaning in life and search for meaning in life. Psychological distress is likely to decrease in the presence of a high level of satisfaction with life and meaning. Moreover, satisfaction with life and search for meaning in life significantly mediated the association between COVID-19 obsession (*z*=-3.507, *p* < *0*.0001 and *z* = −2.632, *p* < 0.001 respectively).

**Conclusion:** The present study showed that life satisfaction and search for meaning in life may play a significant role in decreasing psychological distress during the COVID-19 pandemic.

## Introduction

In December 2019, soon after the outbreak of coronavirus disease 2019 (COVID-19) among individuals in China, the number of cases has increased daily and is updated minute-by-minute by global media, as well as national and international health organizations (such as World Health Organization). In addition to infection rates, information is also provided by such outlets related to its spread, incubation period, lethality symptoms, clinical outcomes, and survival rates (Corman et al., 2020). In any community crisis, individuals try to stay informed. However, when content from official channels is inadequate or irregularly circulated, or when individuals have ambiguity, this can lead to a high level of anxiety among healthy individuals and those with pre-existing mental health problems (Mowbray, 2020).

Beyond the impact on physical well-being, researchers and practitioners have shown concern over COVID-19's impact on mental health (Cao et al., 2020; Pfefferbaum and North, 2020), as seen among previous epidemics, such as the severe acute respiratory syndrome (SARS) outbreak in 2003 (Cava et al., 2005; Mak et al., 2009) and the novel influenza (H1N1) virus outbreak in 2009 (Pfefferbaum et al., 2012). Although research related to psychological distress among patients with COVID-19 is increasing, many emphasize that fear and uncertainties are often driven by a distorted perception of risk among vulnerable populations (Torales et al., 2020).

The current pandemic is affecting the overall well-being of individuals globally. Psychological issues such as fear, stress, denial, anxiety, anger, depressive symptoms, and insomnia are aggravating and further hinder the fight against COVID-19 (Kang et al., 2020). A recent study from Beijing, China asserted that after the outbreak of COVID-19, the prevalence of major depression and post-traumatic stress disorder (PTSD) ranged from 4 to 7% in the general population (Mowbray, 2020). Exposure to adverse and stressful life events, like the current pandemic or other disasters, tends to produce anxious thoughts and in some cases obsessive thoughts that are recurrent and unwanted, causing distress and are associated with other stress-related disorders (e.g., obsessive-compulsive disorder, PTSD, depression, insomnia, and other anxiety-related symptoms) (Marroquín et al., 2020).

Keeping updated with information related to COVID-19 can help individuals to stay safe. However, obsessing too much about the disease may contribute to unhealthy consequences (Taylor, 2019). Researchers suggest that during a pandemic, individuals can exhibit anxiety and fear-related distress, along with accompanying frequent and persistent thoughts, about getting infected or contaminated (Taylor et al., 2020) even over the mild condition that may mimic symptoms of the common cold, leading to compulsive checking and seeking reassurance (Banerjee, 2020). Similarly, fear of unemployment, intrusive thoughts about economic and career crisis can further lead to anxiety and depression in some cases (Taylor et al., 2020). A recent study (Lee, 2020) examined obsession with COVID-19 and identified psychological distress and functional impairment among the general population. The results of the study emphasized that excessive obsessional thoughts about the pandemic were significantly associated with an increased level of anxiety, spiritual crisis, extreme hopelessness, suicidal ideation, and substance abuse.

Empirical research has shown that when experiencing trauma, extreme obsession generates negative emotions. The uncontrollable frequent and persistent thoughts tend to strengthen fear, anger and/or depressive symptoms. These thoughts may facilitate loss of control, low self-esteem, and low confidence and sometimes significant disturbance in daily functioning, therefore adversely affecting the overall well-being of an individual (Christ et al., 2020). Consequently, psychological distress induced by these obsessions may further affect life satisfaction.

Life satisfaction includes a conscious cognitive judgment of the general quality of life based on a subjective, unique set of criteria (Pavot and Diener, 2009). Previous data suggest that individuals with higher satisfaction with life exhibit more elevated levels of resilience, self-esteem, and self-confidence (Martínez-Martí and Ruch, 2017), whereas lower satisfaction with life generates stress (Lee et al., 2016), anxiety, and depression (Beutel et al., 2010). Studies have shown that satisfaction with life is fundamentally associated with improved cognitive and social functioning and promoting optimism, social support, gratitude, social relationship, and health-seeking and improving behavior (Trzebiński et al., 2020).

Likewise, studies suggest that in addition to satisfaction with life, having meaning in life is significantly associated with good psychological well-being (Brassai et al., 2011). Meaning in life is defined as a generally stable sense of purpose in life and an accompanying sense of fulfillment (Baumeister, 1991), and a contributor to good psychological health (Zika and Chamberlain, 1992; Brassai et al., 2011). The literature asserts that, in a traumatic context, having meaning in life can help individuals cope well and predict satisfaction with life (Drescher et al., 2012). It can further contribute to post-disaster resilience (Park, 2016). In the present study, meaning in life was assessed in terms of two distinct aspects (i.e., the presence of meaning in life and the search for meaning in life). The presence of meaning in life suggests that individuals have a purposeful life, whereas individuals searching for a meaning in life have not yet found fully found the purpose (Steger et al., 2006; Aftab et al., 2020). Consequently, the presence of meaning in life is generally associated with more positive outcomes, whereas the search for meaning in life is associated with poorer outcomes (Aftab et al., 2020). Meaning in life forms a fundamental variable of different theories of psychological well-being (King and Napa, 1998). Seligman (2002), in his idea of happiness, proposes meaningful life as an ultimate way of achieving satisfaction in life and authentic happiness. Similarly, according to the Positive Emotion, Engagement, Relationships, Meaning, and Accomplishment (PERMA) theory (Seligman, 2011), and the flourishing elements approach (Diener et al., 2010) that promote individual well-being (Hone et al., 2014) psychological distress, including stress, anxiety, depression, dependency, and suicide, tend to arise in the absence of a sense of meaning (Mascaro and Rosen, 2005; McDowell, 2010) and contributes to a low level of life satisfaction (Ryff and Singer, 1998; Ryff et al., 2004).

Empirical research shows that individuals who have meaning in their life or search for one, tend to have good physical and psychological well-being. They have a high level of hope, greater life satisfaction, positive social interaction, increased self-confidence, better self-actualization, and better overall sound quality of life, such as positive affect, high self-esteem, increased optimism, increased hope, happiness, greater curiosity, increased self-actualization, and positive social interaction (Steger et al., 2008b; Steger and Shin, 2010; Brassai et al., 2011).

Moreover, individuals with high levels of meaning in life tend to have lower negative affect, suicidal ideation, and substance abuse (Steger et al., 2008a) and a lesser need for psychotherapy and positive human functioning (Lopez and Snyder, 2011). However, there has been little research on the sense of meaning in life in the context of the COVID-19 pandemic. Trzebiński et al. (2020) proposed that individuals with increased satisfaction and meaning in life are less likely to generate a negative emotional response to the apparent danger of an ongoing pandemic crisis. They suggested that having meaning in life and satisfaction with life can buffer against anxiety and intrusive thoughts produced due to unpredictable threats such as COVID-19.

Subsequent literature has documented the mediating effect of meaning in life on psychological distress along with unwanted and uncontrollable thoughts. These thoughts, followed by stressful events in life can make life appear insignificant and not worth living, and may contribute to severe depression, and persistent thoughts about death (Bergman and Bodner, 2018). Another recent study by Tomaszek and Muchacka-Cymerman (2020) highlighted the mediating role of satisfaction with life on PTSD symptoms and post-traumatic growth triggered by the COVID-19. Their study found that traumatic events such as the pandemic adversely affected post-traumatic growth.

Considering the significance of meaning in life in the context of psychological distress, obsessive thoughts, and effects of COVID-19, the purpose of the present study was to identify direct as well as indirect associations between obsessive thoughts related to COVID-19, meaning in life, effects of COVID-19, satisfaction with life, and psychological distress. Although the mediating role of satisfaction with life and meaning in life has been previously investigated with various psychological variables, they have not been investigated in the context of COVID-19. Therefore, it was hypothesized that life satisfaction and meaning in life would mediate the relationship between obsessive thoughts and psychological distress. Moreover, it was expected that the effects of COVID-19 would likely mediate the association between psychological distress and obsessive thoughts.

## Method

### Participants

The sample size was calculated through G^*^ Power calculator, assuming a low to moderate effect size with 95% CI. The present study comprised 1,002 participants [445 men (44%) and 548 women (56%)] aged between 19 and 45 years (*M* = 24.30, *SD* = 7.29). The participants were recruited from the Punjab province, which occupies 52.95% of the total population of Pakistan (Pakistan Bureau of Statistics, 2017). Being a highly populated province, the ratio of COVID-19 cases remained high during the outbreak of COVID-19. Therefore, the sample was only recruited from the Punjab province. Most participants were aged 19–27 years (76%), followed by 28–36 years (18%) and 37–45 years (6%). Participants' education level varied from matric (Grade 10) to PhD (i.e., matric = 58 (6%), Intermediate [Grade 12] =125 (13%), graduate = 564 (56%), Masters = 192(19%) and MPhil/PhD = 63 (7%). Among these, 80% (*n* = 812) were non-working (e.g., students, housewives), and 20% (*n* = 190) worked in different domains.

### Procedure and Ethics

Participants were recruited via an online survey using convenience sampling. The inclusion criteria were that participants (i) were citizens of Pakistan and living in Pakistan since the outbreak of COVID-19, and (ii) had not been referred to any psychological services or have any physical disability. In the present study, all participants were asked to complete an online survey with no physical or direct contact with researchers. The online survey was developed utilizing *Google Forms* and distributed via social media networking sites (e.g., *Facebook, Instagram, WhatsApp*). The online survey started with details of informed consent and information about the objectives of the study and the contact details of the principal researcher. Participants were assured of confidentially, anonymity and that information provided would only be for academic and research purposes. The study was approved by the research team's university ethics committee.

### Measures

The survey asked for information concerning participants' socio-demographic characteristics, including information about gender, age, education, religion, citizenship, travel history after the COVID-19 pandemic, consultation with psychological services, and physical disability. The study also utilized valid and reliable measures to assess COVID-19 obsession, satisfaction with life, meaning in life, and psychological distress. Urdu is the national language of Pakistan, therefore Urdu versions of all scales were used. Permission to use the scales was obtained from the authors although all of the scales are in the public domain.

#### Assessment of COVID-19 Obsession

Obsession with COVID-19 was assessed using the four-item Urdu Version of Obsession with COVID-19 Scale (OCS-Urdu Version). The OCS is a newly developed and validated instrument (Ashraf et al., 2020) that assesses disturbed and persistent thoughts concerning COVID-19. Sample items include “*I had disturbing thoughts that I may have caught the coronavirus*.” Responses are scored on a five-point Likert scale ranging from 0 (*not at all*) to 4 (*nearly every day over the past 2 weeks*) with total scores ranging from 0 to 16. High scores are an indicator of dysfunctional thinking and intense obsessions and frequent thoughts concerning COVID-19. The scaling format of the OCS is compatible with the DSM-5 Level 1 cross-cutting symptom measure of mental health domains in psychiatric diagnosis. The present study estimated the omega reliability of the OCS as being 0.76.

#### Assessment of Effect of COVID-19 on Life

The extent of the effect of COVID-19 on an individual's life was assessed utilizing a self-constructed statement (i.e., “*To what extent has the COVID-19 pandemic affected your life?”*) ranging from 1= (*not at all*) to 10 (*completely*).

#### Assessment of Psychological Distress

Psychological distress was assessed using the General Health Questionnaire (GHQ-12) Urdu version (Minhas and Mubbashar, 1996). The GHQ-12 (Goldberg and Williams, 1988) is a commonly used measure to assess psychological distress. It comprises 12 items on three dimensions [social dysfunction = six items (e.g., “*Been able to concentrate on what you are doing”*); depression and anxiety = four items (e.g., “*Felt constantly under strain*”); and loss of confidence = two items (e.g., “*Been losing confidence in yourself”*)] with scores ranging between 0 and 36. High scores are an indicator of greater psychological distress. Participants respond to the items on a four-point Likert scale ranging from 0 (*never*) to 3 (*always*). The present study estimated the omega reliability of the GHQ-12 as being 0.76. The subscales were also found satisfactory (social dysfunction = 0.77, anxiety and depression = 0.78, and loss of confidence = 0.77).

#### Assessment of Satisfaction With Life

The five-item Satisfaction with Life Scale (SWLS; Diener et al., 1985; Urdu version: Barki et al., 2020) was used to assess global cognitive judgments of satisfaction with one's life where responses to items (e.g., “*In most ways my life is close to my ideal*”) are rated on a seven-point Likert scale ranging from 1 (*strongly disagree*) to 7 (*strongly agree*). Scores on the SWLS range from 5 to 35, with higher scores indicating higher life satisfaction. These scores can also be further categorized as extremely satisfied (31–35), satisfied (26–30), slightly satisfied (21–25), neutral (20), slightly dissatisfied (15–19), dissatisfied (10–14), and extremely dissatisfied (5–9). The present study estimated the omega reliability of the SWLS as being 0.87.

### Assessment of Meaning in Life

The ten-item Meaning in Life Questionnaire [MLQ; Steger et al., 2006; Urdu version: (cf. Steger, 2020)] was used to assess meaning in life across two dimensions; (i) presence of meaning in life (five items: e.g., “*I understand my life's meaning”*) and search for meaning in life (five items: “*I am seeking a purpose or mission for my life”*). Both subscales are scored separately. Participants respond to items on a seven-point Likert scale ranging from 1 (*absolutely untrue*) to 7 (*absolutely true*), with scores ranging from 5 to 35 on each subscale. High scores indicate more meaning or search for meaning in the individual's life on each subscale. The present study estimated the omega reliability of the MLQ as being 0.92 (as well as 0.87 for the presence of meaning and 0.89 for the search for meaning).

### Statistical Analyses

The sample's characteristics, such as means, standard deviations, frequencies, percentages, skewness, and kurtosis, were calculated using descriptive statistics. Internal consistency (reliability coefficients) of measures were estimated using omega coefficients (McDonald, 1999). Skewness values ranging between ±0.5 and kurtosis less than ±3 demonstrated normal data distribution. Coefficient omegas also fell within the ranges (<0.70) (see Table 1). Parametric bootstrapping analyses were utilized to test the mediational model of satisfaction with life, the effect of COVID-19, and meaning in life as parallel mediators in the association between COVID-19 obsession and psychological distress. In all mediational analyses, significance was determined at 95% (Preacher et al., 2007). In addition, correlation analyses were utilized to examine the significant association between participants' demographic characteristics and study variables. Three mediation models, parallel mediation, and serial mediation, were hypothesized and tested. The parallel mediation model tested the indirect effect of OCS on psychological distress via life satisfaction, the impact of COVID-19, and meaning in life. All variables were standardized before running statistical analyses to make sure of statistical comparability. All mediational analyses were performed by running PROCESS by Hayes (2013) utilizing SPSS v.24. From the PROCESS function, model 4 was applied for parallel mediation assessment, and model 6 for serial mediation paths.

**Table 1:** Relationship and descriptive characteristics of study variables.

**Measures**	**Gender**	**Age**	**Education**	**SWL**	**OC**	**EAC**	**PML**	**SML**	***M(SD)***	**α**	***Skewness***	***K***
Psychological distress	0.159^*^	0.414^***^	0.284^***^	−0.478^***^	0.350^***^	0.201^**^	0.025	0.182^**^	19.84 (5.47)	0.78	0.307	−0.501
1- Social dysfunction	−0.167^*^	0.267^**^	0.181^*^	−0.455^***^	0.270^***^	0.212^**^	−0.185^**^	−0.055	10.13 (2.91)	0.75	0.872	−0.384
2- Anxiety depression	0.195^**^	0.387^***^	0.263^***^	−0.337^***^	0.267^***^	0.119^**^	0.043	0.196^**^	5.97 (2.84)	0.81	−0.462	−0.433
3- Loss of confidence	0.237^***^	0.325^***^	0.267^***^	−0.295^**^	0.316^***^	0.017	0.241^**^	0.281^***^	3.74 (1.83)	0.79	−0.791	−0.522
PM	0.034	0.045	0.006	0.239^***^	−0.194^**^	−0.193^**^	–	–	18.19 (6.70)	0.87	0.385	−0.328
SM	0.030	−0.047	−0.052	0.357^***^	−0.197^**^	−0.263^***^	–	–	18.89 (7.65)	0.89	0.542	−0.572
SWL	−0.023	0.165^*^	−0.199^**^	–	−0.216^**^	−0.267^***^			16.62 (6.49)	0.87	0.988	0.379
OC	0.165^**^	−0.010	0.027	–	–	0.183^**^	–	–	12.23 (3.13)	0.76	−0.943	−0.373
EAC	0.177^*^	−0.025	−0.028	–	–	–	–	–	6.74 (3.00)	–	−0.956	−0.544

* *p < 0.05*,

** *p < 0.01*,

*** *p < 0.001*.

*Gender was coded 1 for Males and 2 for Females; SWL, Satisfaction with Life; OC, Obsessions about COVID-19; EAC, Extent of affect by COVID-19; PML, Presence of meaning in life; SML, Search for meaning in life; SWL, Satisfaction with life; K, Kurtosis*.

### Data Processing and Preliminary Analysis

Upon completion of data collection, Excel data was imported to SPSS format, and descriptive information was replaced with numerical values readable in SPSS. Participants not meeting the inclusion criteria of the study were excluded from the analysis. Next, missing values analysis was perfumed, and cases with 15% or more than missing responses were eliminated from the dataset. In contrast, missing values <15% were replaced with the serial mean method using missing values analysis in SPSS. Outliers were identified and eliminated to make sure of the normal distribution of the data. With <5% of eliminated data, the finalized dataset comprised of 1,002 participants.

## Results

### Correlation Analysis

As expected, the correlation analysis demonstrated a significant negative association between psychological distress and satisfaction with life (*r* = −478, *p* < 0.001), and a significant positive association between psychological distress with the search for meaning in life (*r* = 0.182, *p* < 0.001), effect of COVID-19 on an individual's life (*r* = 0.201, *p* < 0.01), and COVID-19 obsession (*r* = 0.350, *p* < 0.001). In addition, satisfaction with life had a significant positive association with presence of meaning in life (*r* = 0.239, *p* < 0.0001), and search for meaning in life (*r* = 0.357, *p* < 0.0001), and a significant negative association with COVID-19 obsession (*r* = −0.201, *p* < 0.01) and effect of COVID-19 (*r* = −0.267, *p* < 0.01).

### Covariates

In relation to personal characteristics, gender was positively associated with psychological distress (*r* = 0.159, *p* < 0.05), COVID-19 obsession (*r* = 0.165, *p* < 0.01), and effect of COVID-19 on an individual's life (*r* = 0.177, *p* < 0.05) indicating that females reported higher psychological distress, COVID-19 obsession, and perceived effect of COVID-19 on their life compared to males. Age was also positively correlated with psychological distress (*r* = 0.414, *p* < 0.001), and satisfaction with life (*r* = 0.165, *p* < 0.05) indicating that older Pakistani adults reported more psychological distress and satisfaction with life. Moreover, education was positively associated with psychological distress (*r* = 0.284, *p* < 0.001), and negatively associated with life satisfaction (*r* = −0.199, *p* < 0.01) (i.e., those with higher education had greater psychological distress and poorer life satisfaction).

### Parallel Mediation

**Figure 1:**
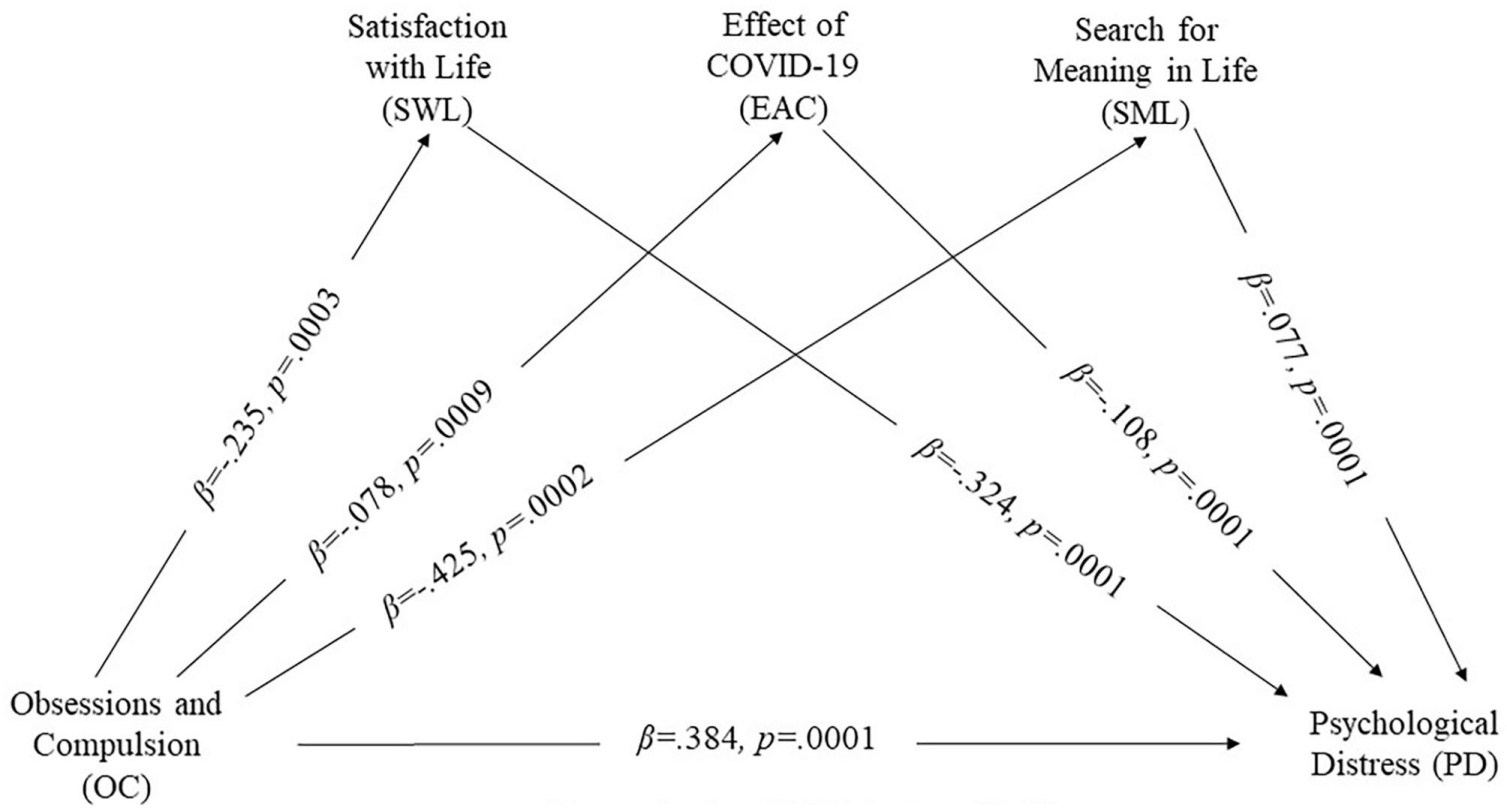
Parallel mediation model.

Findings based on 5,000 bootstrapped samples and controlling for age, gender, and education, showed that the total effect of COVID-19 obsession on psychological distress was significant (total effect = 0.44, SE = 0.050, *p* < 0.0001). The direct effect was also significant (direct effect = 0.389, SE = 0.047, *p* < 0.0001), and indirect effects were present (Figure 1). Overall, two out of three mediators fully mediated the association between obsession and psychological distress (indirect effect = 0.051, SE = 0.021, 95% CI: LL = −0.004, UL = 0.105), illustrating that high-level COVID-19 obsession was associated with low levels of satisfaction with life and meaning in life (see Table 2). These results demonstrated that psychological distress was lower in the presence of high level of satisfaction with life and presence of meaning in life. In addition, satisfaction with life and search for meaning in life significantly mediated the association between COVID-19 obsession and psychological distress (z = −3.507, *p* < 0.0001 and z = −2.632, *p* < 0.001, respectively).

**Table 2:** Standardized indirect, direct effects and total effects of obsession about COVID-19 on psychological distress in parallel mediation model (Model 4).

**Paths**	**Total effect**			**Direct effect (total)**				
	***b (boot SE)***	***95% CI (LL-UL)***	***p***	**Paths**	***b (boot SE)***	***95% CI (LL-UL)***	***p***	**Sobel z**
OC → PD	0.443 (0.050)	0.331–0.531	0.00001	OC → PD	0.389 (0.043)	0.287–0.480	0.0001	
	Direct effects (individual)			Indirect effect of OC on PD				
OC → SWL	−0.235 (0.065)	107–0.362	0.0020	OC → SWL → PD	−0.106 (0.022)	−0.124 to −0.034	0.0001	−3.471^***^
OC → EAC	0.074 (0.030)	0.018–0.137	0.00001	OC → EAC → PD	0.008 (0.005)	0.0001–0.022		1.525
OC → ML	−0.425 (0.136)	−0.693 to −0.156	0.0098	OC → SML → PD	−0.032 (0.013)	−0.065 to −0.011		−2.788^**^
OC → PD	0.436 (0.053)	0.331 to 0.541	0.0001	Total	0.021 (0.027)	−0.004 to 0.105		

** *p < 0.01*,

*** *p < 0.0001*;

*PD, Psychological distress; SWL, Satisfaction with life; OC, Obsessions about COVID-19; EA, Extent of affect by COVID-19; SML, Search for meaning in life; LL, Lower limit; UL, Upper limit; CI, confidence interval; SE, Standard error*.

### Serial Mediation Model

**Figure 2:**
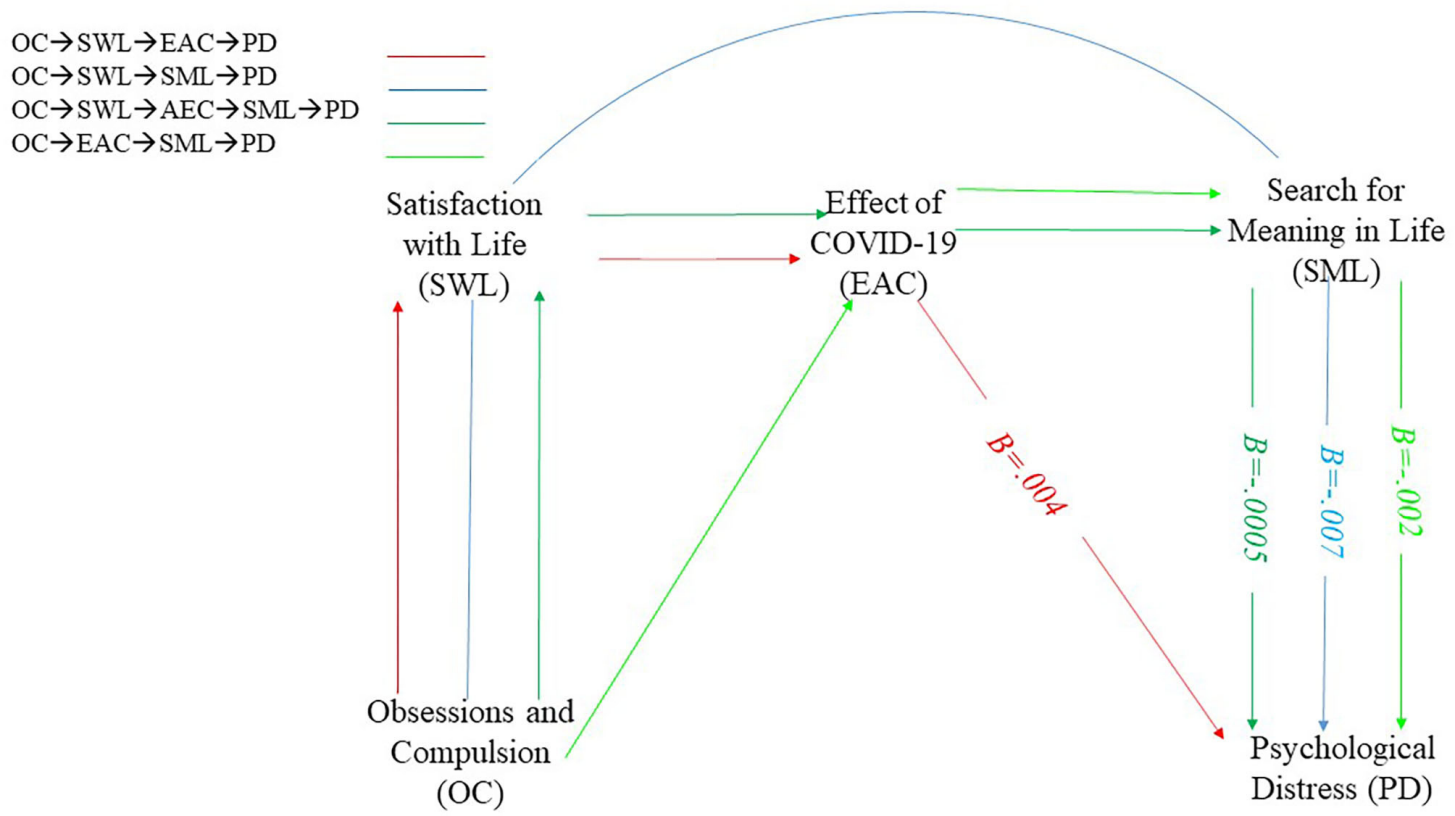
Serial mediation model.

Serial mediation hypothesized an association of mediators (i.e., satisfaction with life, the effect of COVID-19, search for meaning in life) in the relationship between COVID-19 obsession and psychological distress. For example, obsession with COVID-19 could decrease satisfaction with life, which in turn, increases the perception of effect of COVID-19 on one's life and, in turn, could decrease meaning in life, resulting in an increase in psychological distress in life (see Figure 2). However, the results showed no significant mediated associations (see Table 3).

**Table 3:** Standardized mediational effects of obsession on psychological distress in serial mediation model.

**Paths**	**Indirect effects of OC on PD**		**Ratio of indirect effect to total effect**		
OC → SWL → PD	−0.106 (0.02)^*^	−0.124 to 0.032	OC → SWL → PD	0.174 (0.053)	0.076–0.282
OC → SWL → EAC → PD	0.004 (0.001)	0.0001–0.004	OC → SWL → EAC → PD	0.003 (0.002)	0.0003–0.010
OC → SWL → SML → PD	−0.007 (0.003)	−0.014 to −0.002	OC → SWL → SML → PD	−0.012 (0.007)	−0.0328 to −0.006
OC → SWL → AEC → SML → PD	−0.0005 (0.0003)	−0.001 to −0.0001	OC → SWL → AEC → SML → PD	−0.001 (0.007)	−0.003 to −0.0003
OC → EAC → PD	0.006 (0.004)	−0.0001 to 0.020	OC → EA → PD	0.0160 (0.011)	−0.0001 to 0.049
OC → EAC → SML → PD	−0.002 (0.001)	−0.006 to −0.001	OC → EA → SML → PD	−0.010 (0.003)	−0.015 to −0.0001
OC → SML → PD	−0.022 (0.012)	−0.050 to −0.002	OC → SML → PD	−0.102 (0.031)	−0.127 to −0.003

* *p < 0.05 = significant mediation*;

*PD, Psychological distress; SWL, Satisfaction with life; OC, Obsessions about COVID-19; EAC, Extent of affect by COVID-19; SML, Search for meaning in life; LL, Lower limit; UL- Upper limit; CI, Confidence interval; SE, Standard error*.

### Alternate Model Testing

**Figure 3:**
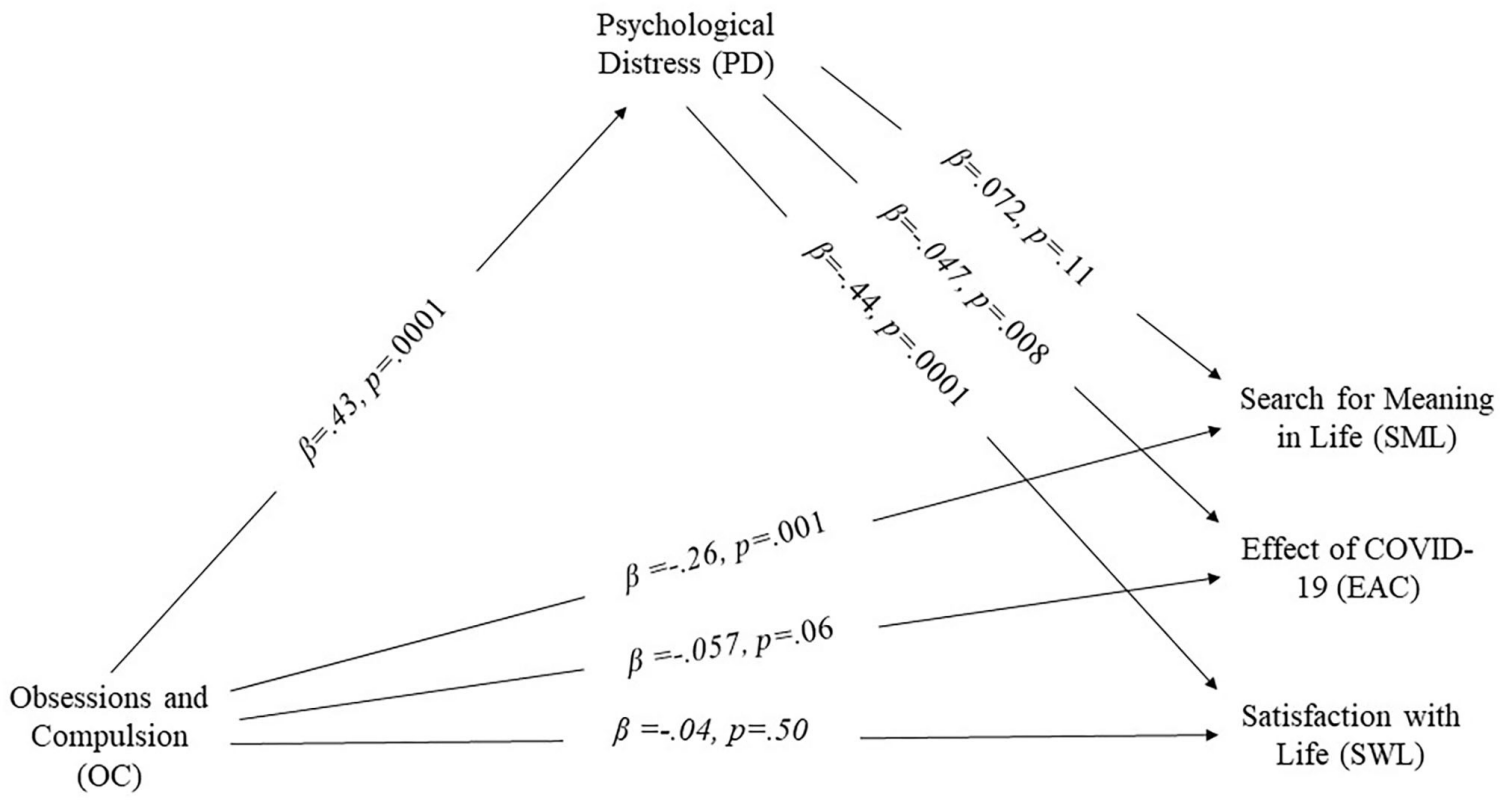
Alternate mediation model.

Along with proposed parallel and serial mediation, alternate mediation models were tested by switching the mediators and outcome variables (i.e., mediating role of obsession with COVID-19 in association of psychological distress with satisfaction with life, the effect of COVID-19, and search for meaning in life). Results showed a significant mediating role of psychological distress in the association between obsession with COVID-19 and satisfaction with life, indicating significant indirect effect (effect = −192, SE = 0.02; z = −6.78, *p* < 0.001). However, in case of the effect of COVID-19 and search for meaning in life, a non-significant mediation effect was found (*z* = −1.507, *p* = 0.121, and *z* = 1.553, *p* = 0.123 respectively) (Figure 3).

## Discussion

Pandemics like COVID-19, not only cause physical health consequences, but are likely to impact the general public's overall quality of life, leading to social dysfunction. Individuals with mental health issues are especially prone to these effects (Banerjee, 2020). The present study investigated individuals' obsessive thoughts toward COVID-19, related psychological distress, and the mediating role of satisfaction and meaning in life on obsessional thoughts and psychological distress. The demographic analysis indicated that most of the sample who reported psychological distress, obsessional thoughts, poor satisfaction with life, and low meaning in life due to the COVID-19 pandemic were female students in young adulthood. Age and education were significantly positively associated with satisfaction in life and psychological distress. These findings concur with the recent literature asserting that young female students are more vulnerable to developing symptoms of various psychological issues during the COVID-19 pandemic, including stress, depression, anxiety, social dysfunction, and post-traumatic stress disorder (Gao et al., 2020). This can be attributed to the fact that when exposed to stressful events, females exhibit differential neurobiological responses, possibly providing the basis for the overall increased susceptibility of developing specific mental disorders among females (Eid et al., 2019). Additionally, previous studies have demonstrated that individuals who are young and students showed more adverse psychological symptoms in the form of emotional distress during the pandemic (Ahmed et al., 2020) due to educational institute closures, delayed exams, low educational efficacy with online classes and distance learning, and lack of in-person social activities (Cao et al., 2020).

Additionally, much research has confirmed the current pandemic's association with poor psychological well-being and aggravated mental health issues among the general population (Lima et al., 2020). For example, a study by Ahorsu et al. (2020) demonstrated that the fear of COVID-19 was positively associated with hospital stress, anxiety, and depression. This might be because of the continued exposure to the news about global fatalities or infection rates and becoming overly familiar with the world crisis over the past few months. This can further lead to worries and irrational or unclear persistent thoughts about the danger of acquiring the virus, and generating clinical fear among the general population (Lin, 2020). This is consistent with the findings in the present study showing a significant positive association between psychological distress, obsessive thoughts, and the effect COVID-19 on individual's lives.

The extant literature emphasizes that satisfaction and meaning in life are associated with reduced psychological distress related to COVID-19 and increased emotional well-being (Schnell and Krampe, 2020; Tomaszek and Muchacka-Cymerman, 2020). Subsequently, in the present study, satisfaction with life and search for meaning in life were found to be significantly and positively correlated with each other. Additionally, psychological distress (assessed as anxiety, depression, social dysfunction, and low level of confidence) in the present study, was significantly negatively associated to satisfaction with life, and positively associated with search for meaning in life. This was also consistent with prior studies suggesting that experiencing stressful life events can adversely affect the person's overall psychological well-being (McGee et al., 2018). Although research studies highlighting the critical impact of COVID-19 on satisfaction with life and meaning in life are few, a Turkish study reported that effects of COVID-19 could cause a high level of distress, depression, and anxiety, leading to a decreased level of satisfaction with life (Satici et al., 2020). Empirical evidence from the present study found that even after controlling for variables (such as gender, age and education), COVID-19 had a direct negative effect on individuals' life satisfaction. This finding is also consistent with the psychological impact of SARS on subjective satisfaction with life (Maunder et al., 2006; Lau et al., 2008). Similarly, as established, stress results from negative, stressful, and harmful events, so having purpose and meaning in life can lead to improved overall health. Studies have demonstrated that having purpose and meaning in life can mediate the negative impact of stressors on an individual's life (Krause, 2007).

Moreover, the present study's findings suggested a negative association between presence of meaning in life and search for meaning with perceived effects of COVID-19 and obsessive thoughts. This finding is in line with that of a past study (i.e., Nicomedesa and Avila, 2020) which found that a higher level of meaning in life was associated with lower levels of panic and anxiety, which in turn facilitated more deliberate and rational behavior and thoughts during the pandemic. This indicates that having meaning in life can provide a secure existential foundation allowing individuals to consider their stressors as more of a worthy challenge rather than a trauma. Consequently, having meaning in life can serve as a source of motivation and compass, irrespective of temporary loss of identity in times of crisis. On the other hand, failure to deal with the difficulties in finding meaning, occurring as a result of stressors, can cause high distress and prevent constructive coping or even lead to self-harming behavior (Schnell and Krampe, 2020).

As expected, the results of the present study showed that satisfaction and search for meaning in life significantly mediated the relationship between psychological distress and obsessive thoughts concerning COVID-19 after controlling for factors (such as age, gender, and education) in parallel mediation analysis. The study showed the significant total effect of obsessive thoughts and psychological distress. More specifically, it showed substantial direct impact and the presence of an indirect effect, emphasizing that level of obsessive thoughts concerning COVID-19 tends to decreases in the presence of a high level of satisfaction and meaning in life. The first two elements, satisfaction, and meaning in life, relate to an individual's self. It can be expected that a high level of meaning in life and enhanced satisfaction with life, can serve as a psychological buffer against harmful and distressing events of life. An increasing number of studies report that elevated level of meaning in life and satisfaction with life are significantly associated with better mental and physical health and the various facets of improved cognitive and social functioning (e.g., Hicks and Routledge, 2013, Batthyany and Russo-Netzer, 2014). The evidence suggests that satisfaction with life equips individuals with psychological stability that in turn helps them in dealing with life's challenges effectively.

Previous research (e.g., Lee, 2020; Trzebiński et al., 2020) has shown that constant thinking about COVID-19 for up to several days can lead to psychological distress (so much so that an individual dreams about it and repetitively, has disturbing thoughts that they may have become infected and/or may have come in contact with an infected person). Although these behavioral patterns are natural cognitive responses in the short-term, they can lead to distressing thought patterns and symptoms of clinical anxiety if they are more persistent (American Psychiatric Association, 2013). Consequently, these thought patterns are considered to be maladaptive, leading to functional impairment. They are associated with other negative behaviors, including drug/alcohol abuse and suicidal ideation (Lee, 2020), further leading to diminished satisfaction and meaning in life (Trzebiński et al., 2020). However, in the present study, no significant causal serial mediation path, including satisfaction with life, the effect of COVID-19, and meaning in life, was established.

It should also be noted that when testing alternate models of mediation (i.e., switching the mediators with outcome variables), a significant mediating role of psychological distress in the association between the obsession with COVID-19 and satisfaction with life was found. This concurs with the recent findings of a Turkish study (Satici et al., 2020) which reported a significant role of fear of COVID-19 on life satisfaction via different psychological distress measures (i.e., depression, anxiety, and stress). Additionally, fear of COVID-19 has been reported to be a strong indicator of obsession and compulsion in the specific context of the COVID-19 pandemic (Ji et al., 2020).

## Limitations

Although the present study provided an insight into the understanding of life satisfaction and meaning in life in the context of obsession concerning COVID-19 and psychological distress, the research is not without limitations. The study utilized self-report measures to assess all study variables, which are subject to well-known methods biases. However, the psychometric scales utilized in the present study have been widely employed across diverse samples (Grant et al., 2003; Hammen, 2008). In future research, semi-structured interviews may provide more in-depth information concerning the study variables and the level of threat associated with these stressors (Hammen, 2008). More specifically, interviews concerning COVID-19 obsession and psychological distress may help establish the timing of onset and duration of these thoughts and behaviors about the variations in satisfaction with life and meaning in life. Finally, these findings warrant replication to ensure that these findings generalize to other samples of adults in other countries and cultures. Longitudinal studies are needed to assess the bidirectional mediational effects between satisfaction with life and psychological distress because the present study could not determine causal effects. Another limitation was recruiting participants from just one of the country's five provinces in Pakistan. Although the Punjab province comprises more than half of the country's total population and is well known for cultural diversity, recruiting participants from the other provinces should be considered for future studies as this would broaden the generalizability of the findings.

## Conclusion

The findings of the present study suggest that a high level of satisfaction with life and having meaning in life can serve as a significant buffer against uncontrollable obsessive thoughts and considerable psychological distress caused by the current COVID-19 pandemic. Given the adverse effect of prolonged obsessive thoughts and related psychological distress on satisfaction and meaning in life, it is essential to enhance individuals' comprehension regarding the direct and indirect pathways underlying these associations. This is so that specific prevention or intervention plans can be developed, helping to improve satisfaction and meaning in life, and improve the overall well-being of an individual.

## Data Availability Statement

The raw data supporting the conclusions of this article will be made available by the authors, without undue reservation.

## Ethics Statement

The studies involving human participants were reviewed and approved by Institutional Review Board, Department of Humanities, COMSATS University Lahore. The patients/participants provided their written informed consent to participate in this study.

## Author Contributions

FA conceptualized the idea, designed methodology, collected, and analyzed data. GZ reviewed literature and discussion, and collected data. AN and AA collected data. AN and MG contributed in conceptualizing the idea and revising the manuscript. All editing at all stages of the manuscript was done by MG. All authors contributed to the article and approved the submitted version.

## Conflict of Interest

The authors declare that the research was conducted in the absence of any commercial or financial relationships that could be construed as a potential conflict of interest.
